# 1090. Does calculation method matter for targeting vancomycin AUC?

**DOI:** 10.1093/ofid/ofab466.1284

**Published:** 2021-12-04

**Authors:** Jack Chang, Dhara Patel, Kimberly C Claeys, Marc H Scheetz, Emily Heil

**Affiliations:** 1 Midwestern University, Downers Grove, Illinois; 2 University of Maryland School of Pharmacy, Baltimore, Maryland; 3 Midwestern University/Northwestern Memorial Hospital, Chicago, IL; 4 University of Maryland, Baltimore, Maryland

## Abstract

**Background:**

Recent vancomycin (VAN) guidelines recommend targeting an area under the curve (AUC) concentration of 400-600 for treatment of methicillin resistant *Staphylococcus aureus* infections. Multiple strategies for calculating AUC exist, including first order pharmacokinetic (foPK) equations and Bayesian models. Most clinical applications of foPK assume unchanged patient status and project ideal administration times to estimate exposure. Bayesian modeling provides the best estimate of true drug exposure and can incorporate changing patient covariates and exact doses. We compared two commonly used foPK methods to Bayesian estimates of VAN AUC.

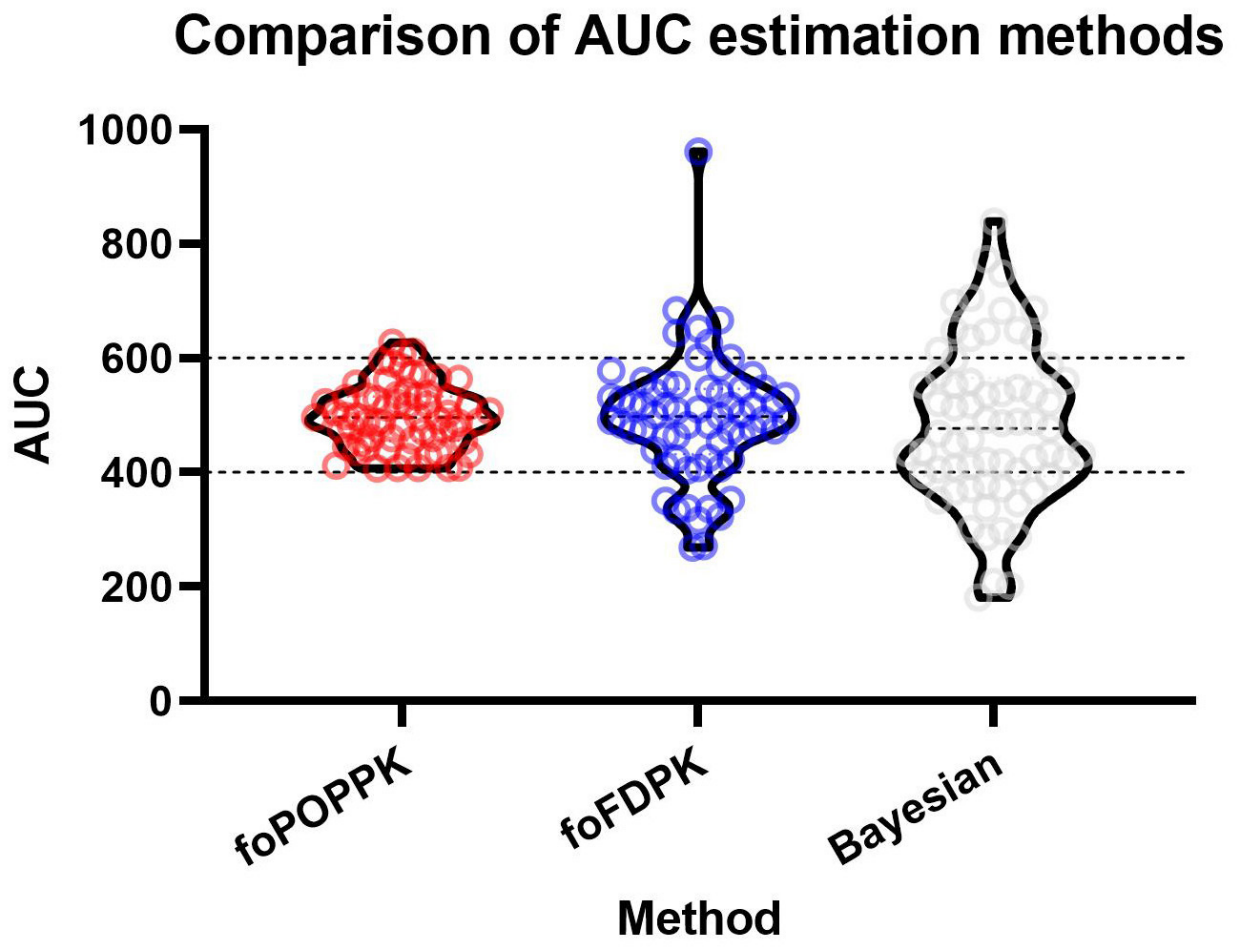

Graphs depict calculated AUCs using the three different methods: 1) Population PK estimated (foPOPPK) 2) Two-level first dose estimated (foFDPK) 3) Bayesian estimated.

**Methods:**

First order equations were performed using population PK estimates (foPOPPK) to estimate steady state (SS) AUC and initial doses. Two concentrations after first dose were used to estimate SS AUC (foFDPK). A 2-compartment Bayesian model allometrically scaled for weight and adjusted for creatinine clearance was used to determine 24-48 hour AUCs. Differences between AUCs were compared using a mixed-effects analysis, and correlation of foPK equations to Bayesian estimates was described using Spearman’s correlation. Patient results from each method were classified as below (< 400), within (400-600), or above ( >600) targets.

**Results:**

65 adult patients were included. The median and IQR for calculated AUCs using foPOPPK, foFDPK, and Bayesian methods were 495.6 (IQR: 76.6), 498.2 (IQR: 107.4), and 472.1 (IQR: 177.9), respectively with p >0.65 for both foPK methods vs. the Bayesian method. AUCs predicted by foPK equations were poorly correlated with Bayesian AUCs (Spearman’s rho= -0.08, p=0.55), while AUCs from foFDPK better correlated with Bayesian AUCs (Spearman’s rho= 0.48, p=0.00). AUCs were within, above, and below target for 54%, 20%, and 26% for the Bayesian model; 95%, 5% and 0% for foPOPPK; and 74%, 12%, and 14% for foFDPK. foPK AUC estimates concurred with Bayesian estimates only 52% of the time.

**Conclusion:**

AUCs calculated by the three methods did not differ on average, but dosing recommendations for foPK at the patient level varied substantially compared to the Bayesian method. This difference is because Bayesian estimation incorporates actual patient exposures while foPK equations rely on idealized dose timing to predict AUCs.

**Disclosures:**

**Kimberly C. Claeys, PharmD**, **GenMark** (Speaker’s Bureau) **Marc H. Scheetz, PharmD, MSc**, **Nevakar** (Grant/Research Support)**SuperTrans Medical** (Consultant)**US Patent #10688195B2** (Other Financial or Material Support, Patent holder)

